# Beyond Cognition: Experts’ Views on Affective-Motivational Research Dispositions in the Social Sciences

**DOI:** 10.3389/fpsyg.2018.01300

**Published:** 2018-07-30

**Authors:** Insa Wessels, Julia Rueß, Lars Jenßen, Christopher Gess, Wolfgang Deicke

**Affiliations:** ^1^Institute for Psychology, Humboldt-Universität zu Berlin, Berlin, Germany; ^2^bologna.lab, Humboldt-Universität zu Berlin, Berlin, Germany; ^3^Department of Education Studies, Humboldt-Universität zu, Berlin, Berlin, Germany

**Keywords:** research competence, affective-motivational research dispositions, research-based learning, higher education, expert interview

## Abstract

Research competence (RC) as a key ability of students in the social sciences has thus far been conceptualized as consisting primarily of cognitive dispositions. However, owing to its highly complex and demanding nature, competence in conducting research might require additional affective and motivational dispositions. To address this deficiency in the literature, first, we conducted a qualitative interview study with academic experts (*N* = 16) in which we asked them to identify challenging research situations and the affective-motivational research dispositions needed to cope with them. We employed a subsequent online rating (*N* = 27) to evaluate the situations and dispositions that had been identified. The resulting affective-motivational facet of RC encompasses six challenging situations that are often encountered and nine dispositions that are necessary to successfully conduct research in the social sciences and may be used to both inform and evaluate research-based learning. The interview-based approach may serve as an exemplary procedure to postulate affective-motivational facets of competence models.

## Introduction

A central aim of higher education is to help students acquire research competence (RC; e.g., British Academy, 2012), and this aim is reflected in the curricula of study programs. The debate on how to correctly teach RC to students has thus gained increased attention (Lewthwaite and Nind, 2016; Roberts, 2016). In the social sciences, a range of research method courses and research-based study projects (e.g., undergraduate research opportunity programs; John and Creighton, 2011) are aimed at equipping students with the competences that are necessary for understanding and conducting research.

Research competence enables students to write final theses and to graduate but is also deemed important for their subsequent professional careers. Research-intensive occupations in the fields of market-, social-, and evaluative research require the ability to conduct research in a self-regulated manner (e.g., Russ-Eft and Preskill, 2009). Other professionals (e.g., teachers and psychologists) are increasingly asked to employ evidence-based thinking and to integrate scientific findings into their daily praxis (Levant et al., 2006; Slavin, 2008).

Accordingly, RC can be understood as the ability to produce research (“engagement in research”; Borg, 2010, p. 391) and the abilities to understand and apply research results (“engagement with research,” ibid.). For the purpose of this paper, the term RC denotes the ability to conduct research in a self-regulated manner (“engagement in research”). This means that students have the competences that are required to successfully complete a classical research cycle, ranging from developing a question to interpreting and communicating the results.

There is wide agreement that equipping students with RC constitutes a central objective of social scientific study programs. However, existing conceptualizations might be incomplete with respect to the extent to which they fully capture the challenges involved in successfully completing a research project.

Existing models of RC focus on cognitive aspects of research and conceptualize RC as primarily encompassing methodological knowledge and skills (Thiel and Böttcher, 2014; Gess et al., 2017). These models and the test instruments that are based on them can help in the capturing and evaluation of students’ RC. However, a focus on cognitive dispositions might render a model incomplete for explaining performance (Blömeke et al., 2015). The highly complex and demanding nature of research might require specific affective and motivational factors. When students engage in research, they often experience emotional unsettlement, especially worry and nervousness (Rand, 2016), and they can be left feeling as though they are facing manifold uncertainties (Delamont and Atkinson, 2001). Conducting research requires self-regulated learning with “students fluctuating between chaos (frustration and disorientation) and cosmos (structures they themselves constructed)” (Silén and Uhlin, 2008, p. 463).

While there is some recognition that the affective-motivational factors involved when students conduct research constitute an important facet of students’ RC (Lei, 2010), a comprehensive description of the nature of these affective-motivational dispositions is missing. Therefore, the purpose of this study is to expand existing conceptions of RC by shedding light on the challenging situations that students face when conducting research and identifying the necessary affective-motivational research dispositions that have been mentioned anecdotally but never comprehensively described.

## Background

### The Affective-Motivational Facet of Competence

There is a long-fought debate that spans the field of educational science on whether competence is constituted solely by cognitive aspects or whether affect and motivation play roles as well. Commonly, the cognitive domain includes an individual’s declarative and procedural knowledge (e.g., skills such as problem-solving strategies and domain-specific knowledge; Weinert, 2001). The affective-motivational domain encompasses beliefs and feelings about the situation or task at hand. These commonly include self-efficacy, interest, achievement goals, and perceived task values (Lau and Roeser, 2002). Weinert (2001) argues for a holistic stance and states that competence “includes all those cognitive, motivational, and social prerequisites necessary and/or available for successful learning and action” (p. 51). Blömeke et al. (2015) claim that “competence involves complex cognitive abilities along with affective and volitional dispositions to work in particular situations” (p. 6). In their view, performance emerges from cognitive and affective-motivational dispositions and situation-specific skills, such as the perception and interpretation of a situation.

Despite these and other theoretical views arguing that competence cannot be reduced to its cognitive dimension (Rychen and Salganik, 2003), many competence models that have been specified for different contexts have addressed only cognitive aspects. These models have often made reference to an article by Koeppen et al. (2008), who defined competence as “context-specific cognitive dispositions that are acquired and needed to successfully cope with certain situations or tasks in specific domains” (2008, p. 62). However, the same authors also stated that when researchers model competence in different domains, they often consider only cognitive dispositions for “pragmatic reasons” (Fleischer et al., 2013) because cognitive competence models are easier to operationalize and assess than those that incorporate non-cognitive aspects as well (Zlatkin-Troitschanskaia and Seidel, 2011). Thus, there seems to be a gap between theoretical views on what competence is and the work that is done to develop competence models: Whereas, from a theoretical perspective, competent performance requires both cognitive and affective-motivational dispositions, the latter are often disregarded in competence models in order to simplify them.

The same can be observed when referring specifically to the domain of RC. Existing models of RC tend to focus on the cognitive dispositions that are necessary to conduct research (Thiel and Böttcher, 2014; Groß Ophoff et al., 2015; Gess et al., 2017). However, a number of studies have described the spectrum of emotions that students experience when they conduct research. Among these are intellectual confusion, emotional unsettlement, worry (all by Rand, 2016), anxiety (Onwuegbuzie and Wilson, 2003), feelings of isolation (Love et al., 2007), the feeling of being “stuck,” disappointment (both by John and Creighton, 2011), and joy about new findings (Fischer et al., 2014). Against this background, it seems shortsighted to assess RC in a purely cognitive way.

### Potential Affective-Motivational Constructs Influencing Research

So far, no comprehensive RC model that includes affective-motivational dispositions exists, but initial clues about which components might constitute the affective-motivational facet of RC can be derived from a discussion of the difficulties students encounter when conducting research.

For students to conduct research, they must shift from passively consuming knowledge to actively creating insight (John and Creighton, 2011). This means they must step into an unknown field with unfamiliar topics and methods that need to be mastered. Open questions and a lack of expertise need to be tackled while advisers offer only limited guidance. As such, conducting research requires strategies for self-regulating one’s learning. Interest and self-efficacy motivate the use of self-regulated learning strategies (Zimmerman, 2000; Sorić and Palekčić, 2009) and are among the central affective-motivational dispositions investigated in research contexts. Research interest, defined as finding interest and enjoyment in conducting different research activities (Bishop and Bieschke, 1998), has been considered in many studies as both a variable of influence and an outcome of research processes. Research self-efficacy, defined as the degree to which a person believes he or she has the competences needed to conduct research (Forester et al., 2004), has been suggested to positively influence beginning and enduring research processes and to predict aspirations for research careers (Adedokun et al., 2013).

While research interest and self-efficacy seem to be helpful dispositions, the “messy, frustrating and unpredictable” (Wellington, 2015, p. 3) nature of research might require additional dispositions. John and Creighton (2011) reported that students struggle in particular with large numbers of setbacks, which induce strong feelings of self-blame. Because frustrations are “integral to the nature of research” (p. 789, ibid.), the ability to handle them well might be another central element of affective-motivational RC. Moreover, the uncertainty and tentativeness inherent to scientific evidence (Bromme and Goldman, 2014) might cause feelings of worry: When a student is tackling a new research topic, not even existing findings can provide the ultimate truth. Students might thus need the ability to find meaning and structure in a sea of uncertainty.

To summarize, many studies have described affective-motivational difficulties from the students’ perspective. However, one deficit of the studies mentioned above is that they have described only individual emotional experiences of students as the students conduct research. Another deficit is that previous studies have often examined only single research dispositions. What is lacking is a systematically derived model of challenging research situations and the affective-motivational dispositions that can help students overcome these challenges.

### The Present Study

Given this state of research, we set out to further explore affective-motivational research dispositions in the social sciences and to synthesize them into a coherent model. Different systematic procedures have been described for postulating new competence models, e.g., through the analysis of requirements and learning goals as stated in national and international curricula (Mayer and Wellnitz, 2013). Alternatively, researchers can employ theoretical psychological-pedagogical considerations to postulate a competence model and empirically validate its structure with factor analysis, as done in the domain of ICT literacy (Zylka et al., 2015). A third approach involves synthesizing the literature to develop competence models that are then empirically tested, e.g., a model of inductive reasoning (Christou and Papageorgiou, 2007).

The application of any of these three approaches would mean that the only aspects that would be considered are those that have already been described elsewhere or are preconceived by the authors. Because affective and motivational aspects are underrepresented in higher educational contexts (Beard et al., 2007), we chose an empirical-exploratory approach to capture new, unexpected aspects and reflect the novelty of the topic. We chose expert interviews as a first method for the present study because they constitute a time-effective way to access the experience-based practical knowledge of the target group (Bogner et al., 2009).

Experts in this context are people who have both extensive knowledge about how to conduct research and many years of experience teaching and supervising students in conducting research. Because affective-motivational dispositions are latent and cannot be directly observed, they have to be inferred from observable behavior (Blömeke et al., 2015). Experts can provide aggregated information on the observed behavior of hundreds or thousands of students while their expertise provides well-founded judgments of what dispositions are necessary for students to successfully conduct research.

In our understanding, the affective-motivational facet of RC consists of research-specific affective-motivational dispositions that functionally refer to the situations and demands of the social scientific research domain (Koeppen et al., 2008). The first central research question that guided our development of a model of affective-motivational RC was thus *(1) Which challenging research situations require dispositions beyond cognitive ones? The second question was (2) Which affective-motivational dispositions are needed to master these situations?*

## Materials and Methods

We employed an exploratory sequential design of the form QUAL > [quan+qual] (Creswell and Plano Clark, 2011) to identify and evaluate relevant research dispositions in a two-step procedure. We applied expert interviews to postulate a model that was then evaluated and refined via an online expert rating.

### Participants

The subsample for the interview study consisted of 16 lecturers (five women) from three German universities (see **Table 1**). We chose these experts on the basis of three selection criteria. Participants (1) had a social-scientific background including political science, sociology, educational science, ethnology, and psychology, (2) represented qualitative, quantitative, and theoretical research, and (3) had substantial experience in the instruction and supervision of students who were conducting research (*M*= 16.01 years, *SD*= 12.81, *min*= 3, and *max*= 46). Their years of experience served as the criteria for expertise in this context. For participants with shared expertise, small sample sizes are sufficient (Romney et al., 1986). However, we did not pre-set the number of participants but conducted the interviews until no substantially new insights were offered after two consecutive interviews (point of saturation).

**Table 1:** Backgrounds of the interview participants.

	Research tradition		
Discipline	Qual (QL)	Quant (QN)	Theoretical (TH)
Educational Science (ED)	2	3	–
Ethnology and Cultural Studies (ET)	2	–	–
Political Science (PO)	–	1	1
Psychology (PS)	–	3	–
Sociology (SO)	2	1	1
**Position/Function**			
Research associate or research management	3		
Post doc	5		
Full professor	6		
Professor emeritus	2		

*Discipline, research tradition, and position were reported separately to avoid identification of individuals. Abbreviations for discipline and tradition are used to denote participants. Example: ED.QN.1 = Educational science, quantitative research tradition, sequential number 1*.

An additional subsample of 27 professors and lecturers in various social science disciplines from nine German universities completed the subsequent online expert rating. Expertise in judging different student RCs was ensured by their position as a full professor or their membership in an advisory network concerned with research-based learning. The experts in the online rating had a moderate range of experience in supervising students who were conducting research of *M*= 13.25 years (*SD*= 10, *min*= 1.5, and *max*= 37). Experts were contacted via e-mail.

### Procedure and Analysis

#### Interviews

We conducted semi-structured interviews to optimally extract the experts’ contextual knowledge (Meuser and Nagel, 2002). The first part of the interview was based on the Critical Incident Technique (Flanagan, 1954): We asked the experts to describe individual students who handled the research process particularly well or poorly. Possible contexts to think about were students writing their final theses, conducting study projects, or working as research assistants at an institute. The second part of the interview was theme-centered (Schorn, 2000) to specifically deepen their thoughts on affective and motivational dispositions. An interview guide (see section “Supplementary Material”) was used as the basis for the interview, but the participants were free to elaborate on any aspects they were asked about. All interviews were conducted by the first author of the study. The interviews were conducted in the offices of the interview participants to provide a quiet and comfortable atmosphere.

The mean duration of the interviews was 00:54 h (*min* = 00:34 h and *max* = 01:28 h). After informed consent was obtained, the interviews were audiotaped and transcribed verbatim. The interviews were conducted and analyzed in German. Selected statements were translated into English for the purpose of this article.

The analysis of the transcripts was based on recommendations made by Meuser and Nagel (2002) and included the paraphrasing and grouping of central text segments. The corresponding author performed the inductive coding process on half of the transcripts. This resulted in a preliminary categorical system.

In order to test the categorical system and its interpersonal application, two raters applied the categories to the remaining transcripts in two steps. In a first step, the corresponding author marked the relevant text segments (based on Schreier, 2012). In a second step, these 283 segments were assigned to the categories by both raters independently. An interrater reliability of Cohen’s kappa = 0.87 demonstrated that the categorical system worked well.

Because the central aim was to identify feasible dispositions, we had several inclusion criteria: Dispositions had to be affective or motivational in nature. We thus excluded descriptions that denoted general personality or that primarily denoted cognitive dispositions. Moreover, dispositions had to be research-specific and could not describe only general academic abilities.

#### Expert Rating

In order to evaluate the model, the relevance of the identified situations and respective dispositions for successfully conducting research were rated on a 4-point Likert scale (ranging from 1 = “not at all relevant” to 4 = “very relevant”) in an online expert rating (Jenßen et al., 2015). Mean scores and standard deviations were calculated to assess the perceived relevance of the dispositions. In addition to this quantitative rating, participants could add comments about any situations or dispositions. These comments were used to sharpen the construct definitions.

On the basis of recommendations by Meuser and Nagel (2002), who discussed the importance of “sociological conceptualization,” the dispositions were then linked to existing concepts from the educational-psychological literature. This provided the theoretical foundation for the model and guaranteed its compatibility with the prevalent scientific discourse.

## Results

**FIGURE 1:**
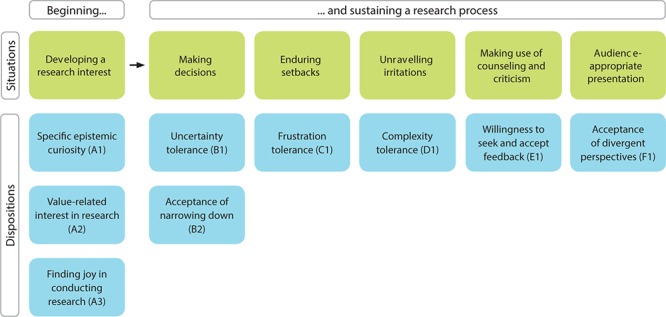
The resulting model of affective-motivational RC. The complete model of affective-motivational RC consists of six challenging situations and nine dispositions needed to master these situations.

In the process of identifying critical situations, it became obvious that the experts’ presentations of the critical situations did not follow the steps of a prototypical research cycle (e.g., literature review and data collection). Rather, the experts named challenges that spanned several steps or recurred throughout the research cycle and the particular dispositions that are necessary to cope with the challenges. In the following section, we describe the situations with their corresponding dispositions one after another (see also **Figure 1**). We jointly present the results from the interview study and the subsequent expert rating.

### Developing a Specific Research Interest (A)

Developing a specific research interest is a crucial situation for commencing and sustaining the research process. It entails the process of transforming an existing personal thematic interest into a research interest. There are different potential origins of a personal interest, including the student’s personal life or thematic stimuli from a lecture. Irrespective of the origin of the interest, it is essential to “tame (the research interest) with regard to content” (SO.QL.1); i.e., a thematic interest has to be adapted so that it is appropriate for use in scientific discourse.

The relevance of this critical situation was rated *M* = 3.73 (*SD* = 0.44). Experts’ qualitative comments furthermore emphasized that it is important for the research topic to be self-selected by the students.

#### Specific Epistemic Curiosity (A1)

According to the experts, curiosity is fundamental for developing a research interest. Almost all experts characterized it as an initial inner urge to investigate a topic or a question that a person finds exciting. They stressed the importance of the inner nature: The students “have to be nuts about something. (…) And it must be their topic, not mine” (SO.QL.1). The urge to find out more about a topic is often connected to a strong desire to unravel the truth. Students do not settle for existing opinions found in the general public or textbooks but want “to say how it really is” (ED.QN.1). Despite the experts’ agreement on the description of this disposition, its exact origin remained unclear.

In line with Litman and Spielberger (2003), the term specific epistemic curiosity was chosen to describe this disposition. The term denotes a directed search for specific knowledge, in contrast to diverse and perceptual curiosity. Its relevance for successfully conducting research was rated *M* = 3.64 (*SD* = 0.56).

#### Value-Related Interest in Research (A2)

In order to turn curiosity about something into a research interest and use scientific rigor to answer a question, students need to value research as an appropriate way to do so. Students embody the motivation to do research when “they realize that they can focus on a certain topic through research” (ED.QL.2). Research thus provides a way to learn more about a topic of interest. Students find research particularly valuable when they realize that it produces results that are relevant for praxis or daily life. According to the experts, these value attributions motivate students to conduct research themselves.

Because this disposition encompasses beliefs about the usefulness of research, we chose the term value-related interest in line with Schiefele (1991). Its relevance for successfully conducting research was rated *M* = 3.16 (*SD* = 0.61).

#### Finding Joy in Conducting Research (A3)

In order to successfully pursue a research interest, it is helpful when research and its individual activities are perceived as enjoyable. Positive emotions regarding research are important for creating a “positive atmosphere” (PS.QN.1), supporting students’ emotional well-being, and improving performance. In addition, joy has a protective effect during the more challenging phases when research tasks that are perceived as less enjoyable need to be completed in order to get back to the tasks that are more enjoyable. As such, joy acts as a driving force to sustain the research process.

We chose the term finding joy in conducting research to describe the positive affect experienced from engaging in different research activities. It resembles the feeling-related component of interest (Schiefele, 1991). Its relevance was rated *M* = 3.44 (*SD* = 0.57). The raters stressed the intrinsic origin of the joy experienced during research: “Students are often far away from secondary motives such as publications, reputation, etc. They do it simply for the sake of doing it.”

### Making Decisions (B)

Students have to make various decisions over the course of the research process, e.g., concerning the feasibility of research questions and matching research designs. Making decisions is difficult for many students, reflected in “decision avoiding techniques” (ED.QN.1) and “jumping from topic to topic” (ED.QN.1). It seems the abundance of alternatives in the research process coupled with a lack of experience poses problems for students, and they try to avoid these problems by employing different escape strategies.

Making decisions was rated as a crucial situation in the research process (*M* = 3.52, *SD* = 0.64). The qualitative comments underscored the prominence of decision making in the research process. It was noted, however, that “wrong decisions provide opportunities for learning.” In this sense, higher education serves as a safe space from which to try one’s hand at research.

#### Research-Related Uncertainty Tolerance (B1)

The process of conducting research was metaphorically described as being similar to entering “a whole new planet” (ED.QL.2) or “a dark chamber” (ED.QL.3). Especially at the beginning of their studies, students often fail to accept the openness of research because they are used to learning clear facts or having somebody guide them. Students begin to struggle during their studies when they “discover that research is actually a lot of the unknown, is full of conflicting opinions, and is ambiguous” (ED.QN.1). The uncertainty arising from the “unknown” quality of research is frequently amplified by the lack of an ultimate truth. To realize that evidence is always only tentative was described as a painful and intimidating experience. Thus, it is necessary to learn to accept and to endure the uncertainty and openness inherent to the research process.

As such, this disposition resembles Dalbert’s (2003) conceptualization of general uncertainty tolerance. We chose the term research-related uncertainty tolerance to capture the research-specific nature of this disposition. Its relevance was rated *M* = 3.48 (*SD* = 0.64).

#### Acceptance of Narrowing Down (B2)

Both the research question and the research process as such need to be narrowed down to become manageable. Choosing and developing a realistic research question constitutes a particular challenge. It is a delicate task for students “to choose research questions that are exciting and original but at the same time workable within the limits of the project” (ET.QL.1). A difficulty in making decisions might be the thought that every decision implies that other possibilities are neglected. Students need to accept that not everything possible or desirable can be worked on because the scope of the project sets limits. It is interesting that the comments indicated that good students in particular seem to have a problem narrowing their focus in order to realistically work on their research.

Another aspect of narrowing one’s focus concerns the ability to terminate the research process. The decision to forgo further exploration and bring a project to a close causes great difficulty. Some students tend to lose themselves in the open field of their research work and greatly enjoy the process of conducting research. These students need to learn to “define their own boundaries” (ED.QL.2). This means that students must adopt a pragmatic stance and stop asking questions at some point.

The acceptance of narrowing down describes the ability to set boundaries for one’s own work within the given context, both when defining the research question and terminating the project. Its relevance was rated *M* = 3.64 (*SD* = 0.56).

### Enduring Setbacks (C)

Enduring setbacks seems to be an inevitable part of conducting research; it might even be at the heart of it: “Research really is (…) an insane amount of frustration. I think I cannot imagine another work place that involves more frustration” (PS.QN.3). For students and senior researchers alike, setbacks might arise from the imponderability of the field, the need to cooperate with a research team or an adviser, or the object of research itself. Other sources of frustration were seen in the relationship between the amount of time spent and the amount of insight created and in recurring feelings of pointlessness when students expressed that they were not uncovering anything new. If these numerous frustrations of exogenous and endogenous origins are not handled well, they might lead to the aborting of the research project.

The relevance of this situation was rated *M* = 3.52 (*SD* = 0.5). One expert emphasized that students had greater trouble enduring setbacks that resulted from interpersonal tension (e.g., with an adviser) than those concerning the project’s contents as such.

#### Frustration Tolerance in the Research Process (C1)

Students need to handle the numerous setbacks that occur during research. When experiencing a setback, “one should not be overwhelmed by feelings of failure such that one does not want to continue” (PS.QN.1). Emotions evoked by setbacks need to be regulated in such a way that a productive continuation of work is possible. Successful students reframe setbacks to advance their current or future research projects by saying, for example, “So that did not work out, but now we at least know what doesn’t work” (PS.QN.3).

In line with the general conceptualization of frustration tolerance, the ability to prevent setbacks from having an action-inhibiting effect is denoted by the term frustration tolerance in the research process. Its relevance was rated *M* = 3.76 (*SD* = 0.51). We confirmed that frustration tolerance is not only about “enduring” but rather about the ability to reinterpret a frustrating situation as something that creates “insight, understanding, and learning.”

### Unraveling Irritations (D)

Several events in the research process can cause astonishment or confusion (e.g., conflicting descriptions in the literature). These affective experiences were summarized as irritations. “If you understand the research process as searching and questioning” (ED.QL.2), then irritations are a natural part of research. Irritations should therefore not be mistaken for setbacks but should rather be seen as “the productive moments when they (the students) realize they were mistaken, they circled around something but did not find it” (ED.QL.2). Irritations can produce insight and help students become acquainted with the field. The beneficial effect of irritations can be unleashed when irritations are explored for their causes and examined for their epistemic value.

The relevance of unraveling irritations was rated *M* = 3.32 (*SD* = 0.68). One rater added that irritations might also create curiosity and provide the motivation to begin a new research process.

#### Complexity Tolerance (D1)

Irritations might have an epistemic value that potentially advances the research process if uncovered. Students thus need a willingness to search for explanations for the irritations they experience. Without this willingness, students do as they are told and stop when things get complicated. By contrast, other students “go further, they do additional analyses, they add another thought (…). Or sometimes the data are very complex, and they nevertheless wade through it” (PS.QN.2). This means these students are not afraid of the complexity that might be added by considering additional thoughts or conducting additional analyses when trying to make sense of irritating situations.

Complexity tolerance thus denotes a constructive stance toward irritations and complexity. We chose the term to show its resemblance to the homonymous disposition described by Radant and Dalbert (2007). Its relevance was rated *M* = 3.52 (*SD* = 0.5). In one of the comments, the importance of the environment was mentioned for developing a tolerance for complexity: It is important that “emerging questions are permitted and evaluated as positive.”

### Making Use of Counseling and Criticism (E)

The presentation or discussion of one’s own research project provides the opportunity for feedback from one’s adviser, research team, or fellow students. The goal is to mutually work with each other’s feedback to advance a project. Feedback can be of a positive, solution-oriented nature, or it can be presented as criticism. Both have the potential to enhance the project’s quality but need to be accepted and understood correspondingly. If feedback and criticism are not requested or not accepted, students may miss an opportunity to improve their work or may become unnecessarily frustrated. The relevance of this situation was rated *M* = 3.56 (*SD* = 0.57).

#### Willingness to Seek and Accept Feedback (E1)

As consultation and criticism are essential for monitoring and improving research work, they should be actively sought. Students who exclusively produce their work “in the isolation of their home offices” (SO.QL.1) are, according to the experts, not the best ones. Instead of working and reflecting on one’s research alone, it is instead more productive for students to re-question the answers they find by seeking the opinions of others. This also requires the courage to put even unfinished research projects up for discussion. Once feedback is sought, it needs to be accepted in a second step. In fact, “there is no point in (…) entering a research context at all if one does not want to learn anything” (ED.QL.2). Accepting feedback implies finding the right balance. On the one hand, students should not be so rattled by criticism that they are intimidated into adopting everything others suggest. On the other hand, they should not be immune to suggestions either.

The relevance of the willingness to seek and accept feedback was rated *M* = 3.44 (*SD*= 0.7). One rater explicitly confirmed the link between seeking advice and performance: “I repeatedly have groups that seal themselves off from feedback. These usually have the worst results.”

### Audience-Appropriate Presentation (F)

While conducting research and after completing it, students need to present their projects to different audiences such as fellow students, researchers, participants in the field, or practitioners. These presentations can be either verbal (e.g., classroom presentations) or written (e.g., theses). Content, demeanor, and speech have to be adapted so that they are appropriate for the target audience and can accommodate perspectives that deviate from one’s own. The relevance of audience-appropriate presentations was rated *M* = 3.08 (*SD* = 0.63). The experts emphasized that the ability to communicate research results provides an important mechanism for self-evaluation.

#### Acceptance of Divergent Perspectives (F1)

Mastering the ability to present in front of different audiences requires students to respect and consider perspectives that do not conform to their own point of view. “One needs to endure different positions – that they exist and that they might be interesting for both sides” (ED.QL.3). In order to make use of different perspectives, it is necessary to “personally adapt to the listener in terms of speech, concepts, and behavior” (PS.QN.3).

We chose the term acceptance of divergent perspectives to denote the ability and willingness to adapt to others. Its relevance was rated *M* = 3.56 (*SD* = 0.57). Comments involved the ability to find a balance between one’s passion and the need for factual presentation to others. Enthusiasm and reflection are not contradictory: “One can be very amazed by one’s own research (…), electrify others, and still act in a very reflected manner.” Accepting perspectives that diverge from one’s own perspective thus means the ability to adapt the contents of one’s research to different audiences and present one’s findings in a factual way without suppressing one’s genuine enthusiasm for the project.

### Excluded Constructs

A number of additional dispositions were proposed in both the interviews and the expert ratings. We had to exclude these on the basis of the inclusion criteria outlined above (see section “Procedure and Analysis”). One example of an excluded construct was knowledge about research ethics, especially for students conducting qualitative studies. While ethical considerations might involve affective aspects and thus be a feasible part of this model, knowledge about how to conduct research in an ethically sound way (e.g., respecting study participants’ wishes to remain anonymous) is knowledge that can be acquired. It should be embedded in a model of the cognitive aspects of RC (as partially realized in Gess et al., 2017).

## Discussion

### The Model

The central concern of this study was to identify challenging research situations and the affective-motivational dispositions needed to master these challenging situations.

The resulting model covers a large breadth of dispositions, ranging from dispositions that concern introspective aspects to dispositions that concern interactions with others. In line with existing research findings, with our model, we acknowledge the importance of interest for successfully conducting research.

Other dispositions, however, were unexpected and had not been conceptualized elsewhere. The disposition we termed “acceptance of narrowing down” is perplexing: While the generation of new knowledge requires interest and curiosity to begin with, our experts also particularly stressed the importance of having the ability to terminate inquiries before they grew too large. RC, therefore, seems to entail a balance of elements: The open and exploratory, as facilitated by complexity tolerance, and the pragmatic and operational, as facilitated by the acceptance of narrowing down.

Overall, the model we developed here goes far beyond the affective-motivational aspects that are usually considered in academic contexts. There are three possible explanations for why the model presented here is different. First, it is possible that research itself is unique in that its challenging nature requires additional dispositions that have not been described in other academic contexts. Interest – that is, among the dispositions that were described previously - might be sufficient for initiating research but might not be enough to master the difficulties encountered during the ongoing research processes. Second, it is possible that the method chosen for the purpose of this study captured different constructs than literature-based procedures. Interviewing experts and specifically asking them to consider students’ emotional and motivational experiences constituted a new step and might have provided a good way to go beyond the usual. Third, common conceptualizations of the affective-motivational facets of competence might focus on only short-term activities such as managing a lesson. Research usually spans several months, thereby increasing the importance of the abilities to regulate affective experiences and sustain motivation. Its long duration might therefore require more or different affective-motivational dispositions.

Because the model was designed to capture affective-motivational dispositions, the individual dispositions had to encompass a strong emotional component, such as feelings of being overwhelmed by uncertainty, or had to function as a motivational force in research contexts. However, it is difficult to fully separate cognitive and affective-motivational aspects – especially in the field of research as a highly cognitive endeavor. Competence models can thus only primarily, but never exclusively, be affective-motivational. Another open question concerns the interplay of cognitive and affective-motivational research dispositions. Only the combination of various cognitive and affective-motivational components are considered to produce competency in a domain (Zlatkin-Troitschanskaia et al., 2015). Previous studies have shown that cognitive variables were stronger predictors of performance, but affective-motivational variables such as engagement demonstrated incremental validity (Lau and Roeser, 2002). How exactly cognitive and affective-motivational dispositions interact to lead to competent performance is, however, unclear (Blömeke et al., 2015). Further studies should thus investigate the interplay between cognitive and non-cognitive facets of RC to provide initial answers to this question for the field of research education.

### Developing Affective-Motivational Research Dispositions

As the dispositions we identified were perceived as very important for mastering critical research situations, whether or not they can be changed is an important question. The general belief is that the dispositions described above can be developed through research participation. Most experts stated that beginning students lack many of the RCs that are needed to be successful in research, but they gradually develop these affective-motivational dispositions through experience.

Research-based learning provides a promising method for facilitating RC development through active engagement in several steps of the research cycle. The potential of research-based learning for strengthening non-cognitive constructs such as research interest and self-efficacy was already shown (Deicke et al., 2014). However, classical teaching formats could also provide enough room to address individual dispositions. For example, lecturers might strengthen uncertainty tolerance by discussing the importance of uncertain results for objective research with their students.

Moreover, gaining knowledge about how affective-motivational dispositions influence students’ research work might help in addressing problems in research education. It is often reported that students are not interested in learning about research (Vittengl et al., 2004). The complex nature of research might be overwhelming and might thus result in decreased interest. Reflecting on challenging situations in the research process might help lecturers foresee difficulties and address these difficulties in their teaching. The proposed model is thought to provide insights into particularly demanding components of the research process that need to be explicitly addressed to prevent negative effects of frustration. Moreover, the model provides a collection of objectives of research-oriented teaching besides the usual knowledge-based learning objectives and thus fills the research gap outlined by Earley (2014).

### Limitations and Future Research

We developed this model on the basis of interviews and expert ratings of students’ research experiences, mainly from professors. We chose the expert-based approach because a small number of professors can provide very valuable information about a large number of students, and the status of the people we interviewed guaranteed valid judgments of what is necessary to be successful. It would be interesting to complement the experts’ views with the perspectives of students who might have different insights into their struggles and different ideas about what is necessary to conduct research in the long term.

Another limitation concerns the generalizability of the results to other disciplines. We conducted the study with a sample of experts from the social sciences because we decided to restrict the sample to disciplines working with the same set of methods, mainly those of empirical social science research. Thus, we were not able to determine whether the dispositions we identified will generalize to other disciplines or are exclusive to social science research. It seems plausible to assume that the ability to handle uncertainty or frustration is important in the natural sciences and the humanities as well, but this needs further investigation. We have already developed set of scales to evaluate students’ affective-motivational research dispositions, and these scales are ready to be used in a range of university settings. Studies employing these scales will provide further insights into the relations between individual dispositions within and across different disciplines.

The range of affective and motivational dispositions mentioned in the interviews shows how demanding it is for students to conduct research, even apart from the cognitive work that has to be carried out. However, these affective-motivational dispositions serve as only a prerequisite for competent research performance. As Blömeke et al. (2015) noted, cognitive and affective-motivational dispositions need to be complemented by a range of situation-specific skills to arrive at competent performance. Performance itself is indicated by observed behavior. For the field of student research as an emotionally challenging field, situation-specific skills could include emotion-regulation skills in frustrating situations (e.g., when a student receives critical feedback on his/her master’s thesis from his/her adviser). These skills manifest themselves in observable coping behavior (e.g., the student follows some of the adviser’s suggestions). However, additional research is needed to expand the understanding of how latent dispositions and situation-specific skills interact in student research contexts.

Altogether, this work constitutes a first study in which research dispositions beyond cognitive ones were systematically explored. It underlines the necessity to consider affective-motivational dispositions for the field of student research and is aimed at fueling the debate on affective-motivational aspects of student learning in contexts of higher education.

## Ethics Statement

This study involved interviews with voluntary participants and did not require ethical clearance by an ethics committee. The study was carried out with informed consent from all participants.

## Author Contributions

IW, JR, and CG contributed at all stages of the research process. WD contributed to the design of the study, the interpretation of the data, and the critical revision of the manuscript. LJ contributed to the data interpretation, the drafting of the manuscript, and its critical revision. All authors have approved this final version for publication.

## Conflict of Interest Statement

The authors declare that the research was conducted in the absence of any commercial or financial relationships that could be construed as a potential conflict of interest.
