# Supplementary material for: Beyond Cognition: Experts’ Views on Affective-Motivational Research Dispositions in the Social Sciences
Source: Front Psychol. 2018 Jul 30;9:1300. doi: 10.3389/fpsyg.2018.01300 (PMC6077320; doi:10.3389/fpsyg.2018.01300)
Supplement: Supplementary file 1 [file Data_Sheet_1.DOCX]

Supplementary Material

Beyond Cognition: Experts’ Views on Affective-Motivational Research Dispositions in the Social Sciences

Insa Wessels^*^, Julia Rueß, Lars Jenßen, Christopher Gess, Wolfgang Deicke

*** Correspondence:** insa.wessels@hu-berlin.de

## Interview Guide

*Introduction of interviewers*

*Information on the purpose of the study*

*Instructions for the interview*

*Data protection and informed consent*

**Section I, narrative: General student research competence**

1. You have a lot of experience in supervising researching students. Is there a student you particularly remember? Why?
2. Do you remember a student who was particularly good at conducting research? What characterized this student?
3. Do you remember a student who was not as good at research? What was this student lacking?
4. Do you remember a student who voluntarily worked more on his/her research work than was demanded? Why do you think this was the case?

**Section II, theme-centered: Motivational aspects of research**

1. Which role does motivation play when students work on a research question or participate in a research project?
2. How do you recognize a particularly motivated student?
3. What motivates students to conduct research in the first place?
4. What is demotivating for researching students?
5. If applicable: Why are some students not motivated to do research?
6. Is students’ research motivation alterable? Can supervisors or lecturers influence the research motivation of their students?

**Section III, theme-centered: Affective aspects of research**

- 1. Do students show emotions when they engage in research? If so, which ones?
  2. Do students’ emotions play a role in the research process? If so, at which points?
  3. Which students’ emotions are important for successful research projects?
  4. Have you ever experienced that (missing) emotions impeded a student research project?
  5. If applicable: how should lecturers or supervisors deal with students’ emotions in the research process?

**Section IV: Summary and open questions**

- 1. When you summarize what we talked about during the interview, what do you think characterizes a research habitus?
  2. Looking at your expectations prior to this interview, did you expect a question I did not pose?
  3. Do you have any further questions or comments?

## *Thank you for your participation!*
